# Ranunculin

**DOI:** 10.1107/S1600536810034847

**Published:** 2010-09-04

**Authors:** Michael Benn, Lois Jean Yelland, Masood Parvez

**Affiliations:** a Department of Chemistry, The University of Calgary, 2500 University Drive NW, Calgary, Alberta, Canada T2N 1N4

## Abstract

In the title mol­ecule {systematic name: (5*S*)-5-[(β-d-gluco­pyranos­yloxy)meth­yl]furan-2(5*H*)-one}, C_11_H_16_O_8_, the five-membered ring is essentially planar, the maximum deviation being 0.0151 (13) Å for the O atom. The six-membered ring adopts a chair conformation with puckering parameters *Q* = 0.581 (2) Å, θ = 9.0 (2)° and ϕ = 39.7 (13)°, and with all of the substituents of the glucoside unit having normal equatorial orientations. The crystal structure is stabilized by extensive O—H⋯O and C—H⋯O hydrogen bonding, resulting in a three-dimensional network.

## Related literature

For background to ranunculin, see: Hill & van Heyningen (1951 ▶); Bai *et al.* (1996 ▶); Benn & Yelland (1968 ▶); Boll (1968 ▶); Camps *et al.* (1982 ▶); Fang *et al.* (1989 ▶). For chemical and spectrometric data for closely related, simple butenolides, see: Perry *et al.* (1996 ▶). For comparison bond distances, see: Allen *et al.* (1987 ▶). For puckering parameters, see: Cremer & Pople (1975 ▶).
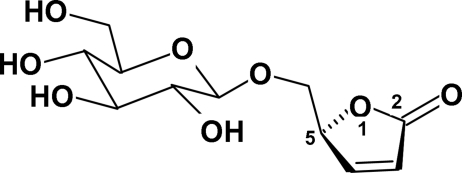


## Experimental

### 

#### Crystal data

C_11_H_16_O_8_
*M* *_r_* = 276.24
Monoclinic, 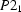

*a* = 5.7944 (4) Å
*b* = 6.9359 (3) Å
*c* = 15.0491 (10) Å
β = 97.895 (2)°
*V* = 599.08 (6) Å^3^
*Z* = 2
Mo *K*α radiation
μ = 0.13 mm^−1^
*T* = 173 K
0.30 × 0.24 × 0.02 mm

#### Data collection

Nonius KappaCCD  diffractometer with APEXII CCD
Absorption correction: multi-scan (*SORTAV*; Blessing, 1997 ▶) *T*_min_ = 0.961, *T*_max_ = 0.997
1926 measured reflections
1133 independent reflections
1112 reflections with *I* > 2σ(*I*)
*R*_int_ = 0.018

#### Refinement

*R*[*F* ^2^ > 2σ(*F* ^2^)] = 0.026
*wR*(*F* ^2^) = 0.069
*S* = 1.04
1133 reflections
184 parameters
1 restraint
H atoms treated by a mixture of independent and constrained refinement
Δρ_max_ = 0.18 e Å^−3^
Δρ_min_ = −0.16 e Å^−3^

### 

Data collection: *COLLECT* (Hooft, 1998 ▶); cell refinement: *DENZO* (Otwinowski & Minor, 1997 ▶); data reduction: *SCALEPACK* (Otwinowski & Minor, 1997 ▶); program(s) used to solve structure: *SHELXS97* (Sheldrick, 2008 ▶); program(s) used to refine structure: *SHELXL97* (Sheldrick, 2008 ▶); molecular graphics: *ORTEP-3 for Windows* (Farrugia, 1997 ▶); software used to prepare material for publication: *SHELXL97*.

## Supplementary Material

Crystal structure: contains datablocks global, I. DOI: 10.1107/S1600536810034847/fl2315sup1.cif

Structure factors: contains datablocks I. DOI: 10.1107/S1600536810034847/fl2315Isup2.hkl

Additional supplementary materials:  crystallographic information; 3D view; checkCIF report

## Figures and Tables

**Table 1:** Hydrogen-bond geometry (Å, °)

*D*—H⋯*A*	*D*—H	H⋯*A*	*D*⋯*A*	*D*—H⋯*A*
O5—H5*O*⋯O8^i^	0.86 (3)	2.17 (3)	2.910 (2)	144 (3)
O6—H6*O*⋯O5^ii^	0.92 (3)	1.94 (3)	2.824 (2)	162 (3)
O7—H7*O*⋯O6^ii^	0.84 (3)	1.84 (3)	2.668 (2)	173 (3)
O8—H8*O*⋯O2^iii^	0.88 (3)	1.96 (3)	2.830 (2)	167 (3)
O6—H6*O*⋯O7	0.92 (3)	2.56 (3)	2.894 (2)	102 (2)

Symmetry codes: (i) 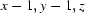; (ii) 
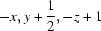
; (iii) 
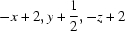
.

**Fig. 1.**
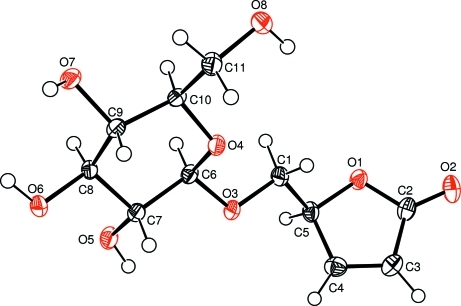
ORTEP-3 (Farrugia, 1997) drawing of the title compound with displacement ellipsoids plotted at 50% probability level.

**Fig. 2.**
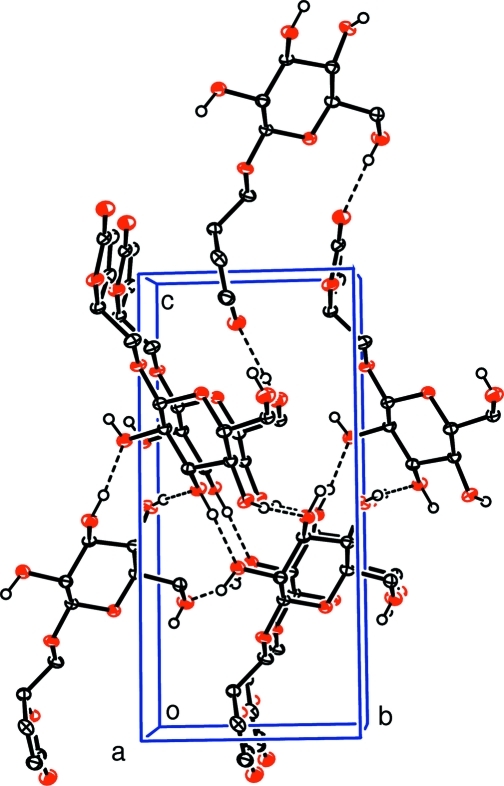
Unit cell packing of the title compound showing intermolecular hydrogen bonds of O—H···O; H-atoms not involved in hydrogen bonds have been excluded.

## supplementary crystallographic information

### Comment

Ranunculin (I) is the glycosidic precursor of the vesicant protoanemonin present in numerous species of *Rannunculaceae* and is especially associated with the burning sensation on chewing leaves of buttercup plants. It was first obtained in crystalline form by Hill and van Heyningen (1951) who established its gross structure and showed that it undergoes enzymatic cleavage by β-glucosidase to yield the aglucone, which underwent easy dehydration to protoanemonin. These processes were shown to occur readily under autolytic conditions (Bai *et al.*, 1996). The *S*-stereochemistry of the dihydrofuranone ring was deduced by Benn and Yelland (1968), and Boll (1968), as shown in the schematic diagram, and later confirmed by synthesis (Camps *et al.*, 1982; Fang *et al.*, 1989). Our sample of (I) was a natural product, and as such had been biosynthesised in the plant (and not made in a laboratory). The only stereoisomer which occurs naturally is the D-isomer, and both that and the anomeric configuration of the glycoosidic bond in (I) were established by cleavage of the glycoside by β-D-glucosidases. The X-ray structure reported here provides the simplest unequivocal proof of that stereochemistry since the chirality follows from that of the D-glucopyranosyl moiety.

The molecular structure of (I) is presented in Fig. 1. The five membered ring, O1/C2—C5, is essentially planar with the maximum deviation of any atom being 0.0151 (13) Å for O1. The six-membered ring adopts a chair conformation with puckering parameters (Cremer & Pople, 1975): *Q* = 0.581 (2) Å, θ = 9.0 (2)° and φ = 39.7 (13)°, with all of the substituents of the glucoside unit having normal equatorial orientations. The 5-membered ring folds back away from the six-membered ring as reflected by the torsion angle C6—O3—C1—C5 -162.86 (17)°. The bond distances and angles are as expected (Allen, 2002). The structure is stabilized by extensive O—H···O and C—H···O hydrogen bonding resulting in a three diemsional network (Table 1 & Fig. 2).

There are two other, closely related, simple butenolides known, though their structures rest on chemical and spectrometric data, i.e., without X-ray crystallographic support. They are the (*5R,6R*) and (*5S,6R*) steeroisomers of 5-([1-β-D-glucopyranosyloxy]ethyl)-2(5*H*)-furanone (Perry *et al.*, 1996); the glycosidic precursor of (*Z*)-5-ethylidene-2(5*H*)-furanone, a homologue of protoanemonin in *Halocarpus biformis juvenile* foliage.

### Experimental

The details of the isolation, and some of the physical properties, of our sample of (I) have been reported previously (Benn & Yelland 1968). Suitable crystals of the title compound for X-ray study were grown from a solution in aqueous ethanol (*ca* 1:20) in the form of plates.

### Refinement

An absolute structure  could not be established reliably because of insufficient anomalous scattering effects. Therefore, 792 Friedel pairs were merged. The H-atoms were located from difference maps and were included in the refinements at geometrically idealized positions with C—H distances = 0.95, 0.99 and 1.00 Å for aryl, methylene and methine type H-atoms, respectively; the positions of hydroxyl H-atoms were allowed to refine freely. The H-atoms were assigned *U*_iso_ = 1.2 and 1.5 × *U*_eq_ of the parent C and O-atoms, respectively. The final difference map was free of chemically significant features.

### Crystal data

**Table:**

C_11_H_16_O_8_	*F*(000) = 292
*M**_r_* = 276.24	*D*_x_ = 1.531 Mg m^−^^3^
Monoclinic, *P*2_1_	Mo *K*α radiation, λ = 0.71073 Å
Hall symbol: P 2yb	Cell parameters from 1560 reflections
*a* = 5.7944 (4) Å	θ = 1.0–30.0°
*b* = 6.9359 (3) Å	µ = 0.13 mm^−^^1^
*c* = 15.0491 (10) Å	*T* = 173 K
β = 97.895 (2)°	Plate, colorless
*V* = 599.08 (6) Å^3^	0.30 × 0.24 × 0.02 mm
*Z* = 2	

### Data collection

**Table:**

Nonius APEXII CCD [APEXII is a Bruker machine - is this a KappaCCD upgraded with an APEXII CCD?]diffractometer	1133 independent reflections
Radiation source: fine-focus sealed tube	1112 reflections with *I* > 2σ(*I*)
graphite	*R*_int_ = 0.018
φ & ω scans	θ_max_ = 25.0°, θ_min_ = 3.2°
Absorption correction: multi-scan (*SORTAV*; Blessing, 1997)	*h* = −6→6
*T*_min_ = 0.961, *T*_max_ = 0.997	*k* = −6→8
1926 measured reflections	*l* = −17→17

### Refinement

**Table:**

Refinement on *F*^2^	Primary atom site location: structure-invariant direct methods
Least-squares matrix: full	Secondary atom site location: difference Fourier map
*R*[*F*^2^ > 2σ(*F*^2^)] = 0.026	Hydrogen site location: difference Fourier map
*wR*(*F*^2^) = 0.069	H atoms treated by a mixture of independent and constrained refinement
*S* = 1.04	*w* = 1/[σ^2^(*F*_o_^2^) + (0.040*P*)^2^ + 0.1617*P*] where *P* = (*F*_o_^2^ + 2*F*_c_^2^)/3
1133 reflections	(Δ/σ)_max_ = 0.002
184 parameters	Δρ_max_ = 0.18 e Å^−^^3^
1 restraint	Δρ_min_ = −0.16 e Å^−^^3^

### Special details

**Table:**

Experimental. NMR data (400 MHz, ^1^H; 100 MHz ^13^C) for a solution in D_2_O containing sodium 3-trimethylsilylpropionate-2,3 - d_4_ as reference: δ_H_ (400 MHz) 7.77 (1*H*, dd, *J* = 1.5 and 5.8 Hz, H-4), 6.3 (1*H*, dd, *J* = 2.1 and 5.8 Hz, H-3), 5.47 (1*H*, m), 4.48 (1*H*, d, *J* = 7.9 Hz, H-1'), 4.30 (1*H*, dd, *J* = 3.2 and 12.2 Hz, H-6 A), 3.95 (1*H*, dd, *J* = 5.8 and 12.2 Hz, H-6B), 3.91 (1*H*, dd, *J* = 2.1 and 12.5 Hz, H-6A'), 3.72 (1*H*, dd, *J* = 5.8 and 12.5 Hz, H-6B'), 3.48 (1*H*, dd, dd, *J* = *ca* 9 Hz H-3'), 3.43 (1*H*, m, H-5'), 3.37 (1*H*, dd, *J* = *ca* 9 Hz, H=4'), and 3.25 (1*H*, dd, *J* = 7.9 and 9.2 Hz, H-2'); δ_C_ 179.2 s (C-2), 158.5 d (C-4), 124.7 d (C-3), 105.6 d (C-1'), 86.9 d (C-5), 78.7 d (C-5'), 78.3 d (C-3'), 75.6 d (C-2'), 72.2 d (C-4'), 71.7 t (C-6), 63.3 t (C-6').
Geometry. All e.s.d.'s (except the e.s.d. in the dihedral angle between two l.s. planes) are estimated using the full covariance matrix. The cell e.s.d.'s are taken into account individually in the estimation of e.s.d.'s in distances, angles and torsion angles; correlations between e.s.d.'s in cell parameters are only used when they are defined by crystal symmetry. An approximate (isotropic) treatment of cell e.s.d.'s is used for estimating e.s.d.'s involving l.s. planes.
Refinement. Refinement of *F*^2^ against ALL reflections. The weighted *R*-factor *wR* and goodness of fit *S* are based on *F*^2^, conventional *R*-factors *R* are based on *F*, with *F* set to zero for negative *F*^2^. The threshold expression of *F*^2^ > σ(*F*^2^) is used only for calculating *R*-factors(gt) *etc*. and is not relevant to the choice of reflections for refinement. *R*-factors based on *F*^2^ are statistically about twice as large as those based on *F*, and *R*- factors based on ALL data will be even larger.

### Fractional atomic coordinates and isotropic or equivalent isotropic displacement parameters (Å^2^)

**Table:**

	*x*	*y*	*z*	*U*_iso_*/*U*_eq_	
O1	0.9146 (3)	−0.1112 (2)	0.96815 (9)	0.0225 (4)	
O2	1.0618 (3)	−0.0493 (3)	1.11025 (10)	0.0309 (4)	
O3	0.4499 (2)	0.0377 (2)	0.79524 (9)	0.0200 (3)	
O4	0.5209 (2)	0.3269 (2)	0.73224 (9)	0.0188 (3)	
O5	0.1227 (3)	−0.0568 (2)	0.63887 (10)	0.0241 (4)	
H5O	0.085 (5)	−0.121 (5)	0.6833 (19)	0.036*	
O6	−0.0241 (2)	0.2692 (2)	0.53178 (10)	0.0208 (4)	
H6O	−0.025 (5)	0.336 (5)	0.479 (2)	0.031*	
O7	0.3667 (3)	0.5126 (3)	0.50561 (9)	0.0215 (3)	
H7O	0.259 (5)	0.591 (5)	0.4895 (18)	0.032*	
O8	0.8805 (3)	0.6376 (3)	0.72096 (10)	0.0277 (4)	
H8O	0.893 (5)	0.563 (5)	0.769 (2)	0.042*	
C1	0.6798 (3)	0.0332 (4)	0.84401 (13)	0.0216 (5)	
H1A	0.7191	0.1601	0.8721	0.026*	
H1B	0.7947	0.0036	0.8030	0.026*	
C2	0.8918 (4)	−0.0724 (3)	1.05518 (13)	0.0225 (5)	
C3	0.6434 (4)	−0.0666 (4)	1.06406 (14)	0.0252 (5)	
H3	0.5794	−0.0486	1.1183	0.030*	
C4	0.5229 (4)	−0.0912 (3)	0.98301 (14)	0.0243 (5)	
H4	0.3578	−0.0895	0.9699	0.029*	
C5	0.6862 (4)	−0.1216 (3)	0.91570 (13)	0.0204 (5)	
H5	0.6609	−0.2517	0.8874	0.025*	
C6	0.4469 (4)	0.1343 (3)	0.71336 (13)	0.0178 (4)	
H6	0.5552	0.0694	0.6765	0.021*	
C7	0.2004 (3)	0.1338 (3)	0.66293 (13)	0.0176 (4)	
H7	0.0906	0.1980	0.6994	0.021*	
C8	0.2093 (3)	0.2433 (3)	0.57590 (13)	0.0167 (4)	
H8	0.2985	0.1652	0.5363	0.020*	
C9	0.3238 (3)	0.4413 (3)	0.59065 (13)	0.0172 (4)	
H9	0.2164	0.5310	0.6170	0.021*	
C10	0.5559 (3)	0.4305 (3)	0.65204 (13)	0.0180 (4)	
H10	0.6738	0.3622	0.6208	0.022*	
C11	0.6423 (4)	0.6302 (3)	0.67948 (14)	0.0218 (5)	
H11A	0.6248	0.7137	0.6256	0.026*	
H11B	0.5424	0.6840	0.7217	0.026*	

### Atomic displacement parameters (Å^2^)

**Table:**

	*U*^11^	*U*^22^	*U*^33^	*U*^12^	*U*^13^	*U*^23^
O1	0.0203 (7)	0.0294 (9)	0.0169 (7)	0.0038 (6)	−0.0002 (5)	0.0024 (6)
O2	0.0327 (8)	0.0329 (10)	0.0233 (8)	0.0036 (8)	−0.0095 (7)	−0.0014 (8)
O3	0.0212 (7)	0.0242 (8)	0.0137 (7)	−0.0011 (7)	−0.0005 (5)	0.0038 (6)
O4	0.0226 (7)	0.0190 (8)	0.0143 (7)	−0.0026 (6)	0.0005 (5)	−0.0001 (6)
O5	0.0317 (8)	0.0223 (8)	0.0172 (7)	−0.0077 (7)	−0.0007 (6)	0.0020 (7)
O6	0.0177 (7)	0.0259 (8)	0.0174 (7)	−0.0007 (6)	−0.0027 (6)	0.0030 (7)
O7	0.0218 (7)	0.0257 (8)	0.0172 (7)	0.0016 (7)	0.0036 (6)	0.0063 (7)
O8	0.0262 (8)	0.0330 (9)	0.0227 (8)	−0.0102 (8)	−0.0010 (6)	0.0034 (8)
C1	0.0200 (10)	0.0262 (12)	0.0175 (10)	−0.0020 (10)	−0.0010 (8)	0.0041 (9)
C2	0.0302 (11)	0.0188 (10)	0.0175 (9)	0.0063 (10)	0.0000 (9)	0.0026 (9)
C3	0.0306 (11)	0.0257 (11)	0.0201 (10)	0.0037 (10)	0.0064 (8)	0.0025 (10)
C4	0.0235 (10)	0.0234 (12)	0.0264 (12)	0.0004 (10)	0.0044 (9)	0.0071 (10)
C5	0.0217 (10)	0.0214 (11)	0.0168 (10)	−0.0001 (9)	−0.0023 (8)	0.0016 (9)
C6	0.0216 (10)	0.0179 (10)	0.0138 (9)	−0.0003 (9)	0.0020 (7)	0.0016 (9)
C7	0.0190 (10)	0.0188 (10)	0.0151 (9)	−0.0014 (9)	0.0025 (7)	0.0003 (9)
C8	0.0152 (10)	0.0207 (10)	0.0135 (9)	0.0003 (9)	−0.0001 (8)	−0.0008 (8)
C9	0.0185 (10)	0.0191 (10)	0.0143 (9)	0.0010 (9)	0.0038 (7)	0.0010 (9)
C10	0.0180 (10)	0.0216 (10)	0.0147 (9)	−0.0004 (10)	0.0038 (8)	0.0017 (9)
C11	0.0253 (11)	0.0206 (11)	0.0189 (10)	−0.0016 (9)	0.0004 (8)	0.0006 (10)

### Geometric parameters (Å, °)

**Table:**

O1—C2	1.361 (2)	C2—C3	1.464 (3)
O1—C5	1.447 (2)	C3—C4	1.331 (3)
O2—C2	1.208 (3)	C3—H3	0.9500
O3—C6	1.401 (2)	C4—C5	1.493 (3)
O3—C1	1.430 (2)	C4—H4	0.9500
O4—C6	1.419 (3)	C5—H5	1.0000
O4—C10	1.443 (2)	C6—C7	1.523 (3)
O5—C7	1.427 (3)	C6—H6	1.0000
O5—H5O	0.86 (3)	C7—C8	1.521 (3)
O6—C8	1.434 (2)	C7—H7	1.0000
O6—H6O	0.92 (3)	C8—C9	1.528 (3)
O7—C9	1.425 (2)	C8—H8	1.0000
O7—H7O	0.84 (3)	C9—C10	1.525 (2)
O8—C11	1.435 (3)	C9—H9	1.0000
O8—H8O	0.88 (3)	C10—C11	1.511 (3)
C1—C5	1.519 (3)	C10—H10	1.0000
C1—H1A	0.9900	C11—H11A	0.9900
C1—H1B	0.9900	C11—H11B	0.9900
			
C2—O1—C5	109.40 (15)	O4—C6—H6	109.9
C6—O3—C1	111.09 (15)	C7—C6—H6	109.9
C6—O4—C10	112.00 (15)	O5—C7—C8	106.92 (16)
C7—O5—H5O	113 (2)	O5—C7—C6	111.72 (17)
C8—O6—H6O	110.7 (18)	C8—C7—C6	106.69 (16)
C9—O7—H7O	105.7 (19)	O5—C7—H7	110.5
C11—O8—H8O	107 (2)	C8—C7—H7	110.5
O3—C1—C5	108.08 (17)	C6—C7—H7	110.5
O3—C1—H1A	110.1	O6—C8—C7	108.63 (16)
C5—C1—H1A	110.1	O6—C8—C9	108.56 (17)
O3—C1—H1B	110.1	C7—C8—C9	112.90 (16)
C5—C1—H1B	110.1	O6—C8—H8	108.9
H1A—C1—H1B	108.4	C7—C8—H8	108.9
O2—C2—O1	120.56 (19)	C9—C8—H8	108.9
O2—C2—C3	130.8 (2)	O7—C9—C10	108.24 (15)
O1—C2—C3	108.66 (17)	O7—C9—C8	107.89 (16)
C4—C3—C2	108.1 (2)	C10—C9—C8	111.93 (17)
C4—C3—H3	125.9	O7—C9—H9	109.6
C2—C3—H3	125.9	C10—C9—H9	109.6
C3—C4—C5	109.8 (2)	C8—C9—H9	109.6
C3—C4—H4	125.1	O4—C10—C11	107.91 (16)
C5—C4—H4	125.1	O4—C10—C9	108.49 (15)
O1—C5—C4	103.91 (16)	C11—C10—C9	110.63 (17)
O1—C5—C1	106.48 (16)	O4—C10—H10	109.9
C4—C5—C1	115.19 (19)	C11—C10—H10	109.9
O1—C5—H5	110.3	C9—C10—H10	109.9
C4—C5—H5	110.3	O8—C11—C10	114.47 (18)
C1—C5—H5	110.3	O8—C11—H11A	108.6
O3—C6—O4	107.90 (15)	C10—C11—H11A	108.6
O3—C6—C7	109.54 (16)	O8—C11—H11B	108.6
O4—C6—C7	109.80 (16)	C10—C11—H11B	108.6
O3—C6—H6	109.9	H11A—C11—H11B	107.6
			
C6—O3—C1—C5	−162.86 (17)	O3—C6—C7—C8	179.11 (17)
C5—O1—C2—O2	−176.8 (2)	O4—C6—C7—C8	60.8 (2)
C5—O1—C2—C3	3.2 (2)	O5—C7—C8—O6	68.0 (2)
O2—C2—C3—C4	176.8 (3)	C6—C7—C8—O6	−172.30 (16)
O1—C2—C3—C4	−3.3 (3)	O5—C7—C8—C9	−171.50 (16)
C2—C3—C4—C5	2.0 (3)	C6—C7—C8—C9	−51.8 (2)
C2—O1—C5—C4	−2.0 (2)	O6—C8—C9—O7	−71.42 (19)
C2—O1—C5—C1	120.06 (19)	C7—C8—C9—O7	168.07 (16)
C3—C4—C5—O1	−0.1 (3)	O6—C8—C9—C10	169.60 (14)
C3—C4—C5—C1	−116.2 (2)	C7—C8—C9—C10	49.1 (2)
O3—C1—C5—O1	−175.41 (16)	C6—O4—C10—C11	−177.97 (16)
O3—C1—C5—C4	−60.8 (2)	C6—O4—C10—C9	62.1 (2)
C1—O3—C6—O4	−61.2 (2)	O7—C9—C10—O4	−169.84 (18)
C1—O3—C6—C7	179.26 (17)	C8—C9—C10—O4	−51.1 (2)
C10—O4—C6—O3	171.64 (15)	O7—C9—C10—C11	72.0 (2)
C10—O4—C6—C7	−69.02 (19)	C8—C9—C10—C11	−169.25 (17)
O3—C6—C7—O5	−64.4 (2)	O4—C10—C11—O8	74.2 (2)
O4—C6—C7—O5	177.31 (17)	C9—C10—C11—O8	−167.27 (16)

### Hydrogen-bond geometry (Å, °)

**Table:**

*D*—H···*A*	*D*—H	H···*A*	*D*···*A*	*D*—H···*A*
O5—H5O···O8^i^	0.86 (3)	2.17 (3)	2.910 (2)	144 (3)
O6—H6O···O5^ii^	0.92 (3)	1.94 (3)	2.824 (2)	162 (3)
O7—H7O···O6^ii^	0.84 (3)	1.84 (3)	2.668 (2)	173 (3)
O8—H8O···O2^iii^	0.88 (3)	1.96 (3)	2.830 (2)	167 (3)
C3—H3···O4^iv^	0.95	2.55	3.413 (3)	151
C4—H4···O1^v^	0.95	2.57	3.504 (3)	168
C8—H8···O7^vi^	1.00	2.37	3.306 (3)	155
C1—H1A···O2^iii^	0.99	2.38	3.289 (3)	153
C10—H10···O6^vii^	1.00	2.43	3.415 (3)	167
O6—H6O···O7	0.92 (3)	2.56 (3)	2.894 (2)	102 (2)
C11—H11A···O7	0.99	2.59	2.986 (3)	104

Symmetry codes: (i) *x*−1, *y*−1, *z*; (ii) −*x*, *y*+1/2, −*z*+1; (iii) −*x*+2, *y*+1/2, −*z*+2; (iv) −*x*+1, *y*−1/2, −*z*+2; (v) *x*−1, *y*, *z*; (vi) −*x*+1, *y*−1/2, −*z*+1; (vii) *x*+1, *y*, *z*.
